# Association between dietary nutrient intake and prevalence of myopia in Korean adolescents: evidence from the 7th Korea National Health and Nutrition Examination Survey

**DOI:** 10.3389/fped.2023.1285465

**Published:** 2024-01-11

**Authors:** Jeong-Mee Kim, Yean Jung Choi

**Affiliations:** ^1^ Department of Visual Optics, Far East University, Eumseong, Republic of Korea; ^2^ Department of Food and Nutrition, Sahmyook University, Seoul, Republic of Korea

**Keywords:** adolescents, dietary nutrients, myopia, prevalence, KNHANES

## Abstract

**Background:**

The rise in myopia prevalence, particularly among adolescents in East Asia, is a cause for concern. While a combination of environmental and genetic factors is understood to contribute to this trend, the role of dietary nutrients is not yet fully clarified.

**Objective:**

To assess the potential association between the intake of specific nutrients and the prevalence of myopia in a large, population-based sample of Korean adolescents.

**Methods:**

Data from 18,077 adolescents (average age: 15.05 ± 1.67 years; 51.7% male, 48.3% female) who participated in the 7th Korea National Health and Nutrition Examination Survey (KNHANES VII, 2016) were analyzed. Refractive error was measured using an auto-refractor-keratometer (KR-8800) without cycloplegia. Dietary intake of 14 nutrients was assessed through a 24-h personalized dietary recall method.

**Results:**

The study revealed a myopia prevalence of 87.6% among the adolescents. Multivariable models adjusted for age, gender, BMI, and other confounding factors indicated that higher intakes of carbohydrates, proteins, cholesterol, sodium, and vitamin B2 were associated with an increased risk of myopia. Conversely, higher intake of vitamin C was found to be associated with a decreased risk.

**Conclusion:**

The findings suggested a potential association between dietary nutrient intake and myopia prevalence in Korean adolescents. While the study did not establish a causal link, the differences in nutrient intake between the myopic and non-myopic groups could indicate that diet plays a role in the development or progression of myopia. Further research is warranted to corroborate these findings and explore the underlying mechanisms.

## Introduction

The incidence of myopia, or nearsightedness, has escalated rapidly over the past few decades. Current projections estimate that by 2050, approximately 50% of the global population will be myopic, with about 10% being highly myopic (1). These figures pose significant public health concerns; it is anticipated that by 2055, the high-risk myopia group will experience a 7–13-fold increase in visual impairments (2). East Asia has been particularly affected, with an alarming rise in myopia rates among children and adolescents (3, 4). The situation is similarly concerning in Korea, where the prevalence of adolescent myopia has recently surpassed 70%, prompting growing attention to the factors that contribute to its onset and progression (5, 6).

Adolescence represents a critical developmental stage characterized by rapid physical growth and increasing educational demands. In the context of Korea's rigorous education system, adolescents face heightened academic pressures and stress (7). This phase also witnesses a shift from outdoor activities to close-distance tasks, including an increased reliance on digital devices (8, 9). While genetic predisposition plays an essential role in myopia onset, the recent surge in prevalence suggests a considerable influence of environmental and lifestyle factors as well (10–13).

Nutritional status, including dietary patterns, can have a profound impact on various aspects of growth and development, including the eye. Recent studies have started to highlight the possible role of diet in the onset and progression of myopia. Nutrients like vitamins, omega-3 fatty acids, and lutein are implicated in a variety of eye-related physiological processes, such as vision maintenance, retinal function, and protection against oxidative stress (14–16). Moreover, nutritional supplements like crocetin and lutein have been reported to ameliorate myopia (17, 18). Given that these nutrients are primarily obtained through diet and are crucial for maintaining eye health, an investigation into dietary factors could offer critical insights into the high prevalence of myopia among Korean adolescents.

Against this backdrop, our study aims to explore the potential association between daily nutrient intake and the prevalence of myopia in adolescents aged 13–18. We utilize data from the National Health and Nutrition Examination Survey to scrutinize this relationship, thereby adding a crucial layer to our understanding of this escalating public health issue.

## Materials and methods

### Study design and population

**Figure 1:**
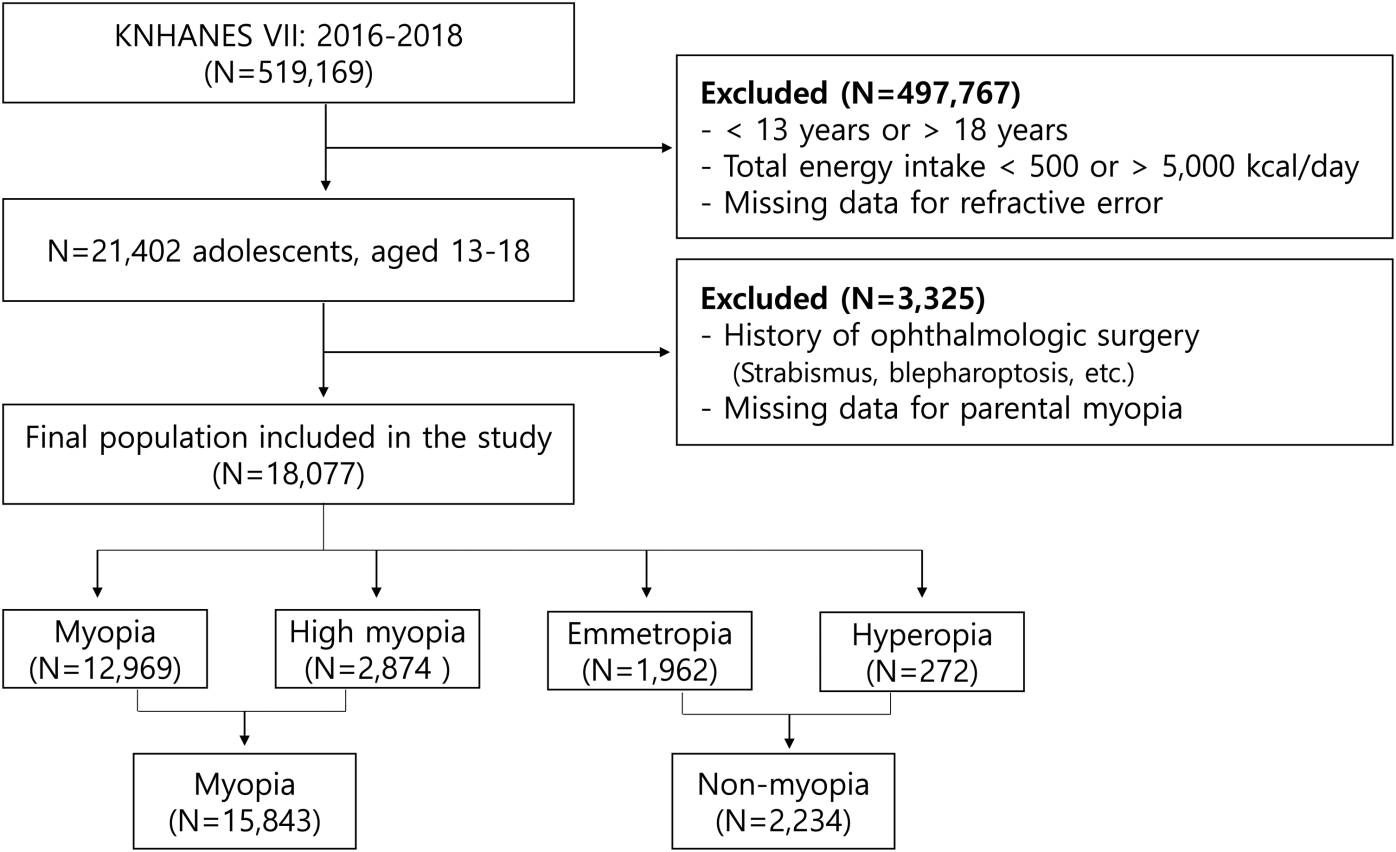
Flowchart for selecting study participants (KNHANES, Korean National Health and Nutrition Examination Survey).

This study utilized data from 519,169 individuals who participated in the 7th Korea National Health and Nutrition Examination Survey (KNHANES VII, 2016), a comprehensive survey conducted by the Korea Centers for Disease Control and Prevention. The primary focus of our analysis was on adolescents aged 13–18 years. To determine the final sample for our study, a multi-step recruitment process was employed. From the total participants of KNHANES VII, we first narrowed down our focus to adolescents aged 13–18. We then applied exclusion criteria to ensure the quality and relevance of the data. Specifically, we excluded cases where the daily total energy intake was either less than 500 kcal or more than 5,000 kcal, as these extremes could indicate reporting errors or atypical dietary patterns not representative of the general adolescent population. Additionally, cases with incomplete refractive error data were excluded (*n* = 497,767). The next step involved excluding adolescents with a history of certain ophthalmic surgeries (such as strabismus or blepharoptosis), as these could be confounding factors in our analysis of nutritional health and its relationship with eye health. Moreover, cases with missing parental information were also excluded to ensure a comprehensive understanding of familial and genetic factors, which are essential in studies involving adolescents (*n* = 18,077). The culmination of this process resulted in a final sample of 18,077 adolescents aged 13–18 years, which is depicted in Figure 1.

### Ocular examination

Refractive errors in both eyes were measured using an autorefractor (KR-8800, Topcon, Tokyo, Japan), without the use of cycloplegic agents. The ocular examinations, including refractive error measurements, were conducted at the survey sites of the KNHANES, which are equipped with necessary ophthalmologic instruments. Spherical equivalent of refractive error (SE) was calculated for each eye, and the analysis utilized the SE value of the right eye. The following formula is used to calculate the SE.

SE=S+(C/2)

(S: Spherical refractive power, C: Cylinder refractive power)

Refractive errors were categorized based on the SE value as follows: myopia (SE ≤ −0.50 D), high myopia (SE ≤ −6.00 D), hyperopia (SE ≥ +0.50 D), and emmetropia (−0.50 D < SE < +0.50 D). For the purposes of this study, these refractive errors were divided into two groups: myopia and non-myopia (comprising emmetropia and hyperopia).

### Covariates

Sociodemographic factors and ophthalmological surgical history were gathered through a survey. Body Mass Index (BMI) was computed using the formula weight (kg)/height (m)^2^ and was categorized into underweight (<18.5), normal (18.5–25), overweight (25–30), and obese (≥30). The survey indeed included data on parental history of myopia, daily hours spent on near work, and the subject's residence, which was classified as either urban or rural. Near work activities primarily involve tasks that require focusing on objects at a close distance, such as reading, writing, and the use of digital devices like smartphones, tablets, and computers.

The KNHANES methodology involves a specialized survey team operating 48 weeks per year across 192 regions. The survey includes on-site health checkups and surveys using a mobile examination vehicle, followed by a nutritional survey conducted at participants' homes by a nutrition researcher within a week of the health assessment. The nutritional survey collected data through face-to-face interviews, focusing on eating habits, food intake frequency, and types of food consumed. An open-ended survey was used to report food intake through a 24-h recall method. The 24-h recall method implemented by KNHANES involved a multi-step process: initially identifying meals and food items, measuring the quantity consumed using tools like cups and spoons, detailed inquiries into food preparation and seasonings, verification of collected food information, and a supplementary survey for additional details. This approach was designed to capture precise dietary data for each participant over a single day. The daily nutrient intake was calculated as the aggregate of all food and nutrient sources consumed during the day. The Korean Dietary Reference Intakes (KDRI) 2020 guidelines were employed to evaluate whether nutrient intake was insufficient or excessive according to age and gender. In this study, the 24-h recall method was used to analyze the daily nutrient intake of 14 components, including carbohydrates, proteins, fats, sodium, and vitamins.

### Statistical analysis

Data were analyzed using the SAS software version 9.4 (SAS Institute Inc., Cary, NC, USA). To account for the complex sampling design of the 7th KNHANES, methods were implemented that considered clustering variables, stratification variables, and weights. Descriptive statistics were expressed as either mean ± standard deviation (SD), frequency, or mean ± standard error (SE). For comparative analysis between myopia and non-myopia groups, the chi-square test was employed for categorical data, while the Analysis of Covariance (ANCOVA) was used for the remaining continuous variables. Both univariate and multivariate logistic regression analyses were performed to calculate adjusted odds ratios and 95% confidence intervals (CIs), aimed at identifying independent risk factors associated with myopia. Three different models were used in the multivariate regression analysis: Model 1 was adjusted for age, gender, and BMI; Model 2 included adjustments for age, gender, BMI, and parental history of myopia; Model 3 accounted for age, gender, BMI, and daily time spent on near work activities. The statistical significance for all tests was set at *p* < 0.05.

## Results

### Characteristics of participants

The study analyzed 18,077 adolescents aged between 13 and 18 years, with a gender distribution of 51.7% male and 48.3% female. The prevalence of myopia within this population was 87.6%, with an average SE value of −3.90 ± 2.28. In terms of Body Mass Index (BMI), the myopic group displayed marginally higher rates of underweight and overweight individuals compared to the non-myopic group. Additionally, parental history of myopia was more prevalent in the myopic group (22.8%) than in the non-myopic group (9.9%). Notably, 70.5% of individuals in the myopic group and 58.1% in the non-myopic group reported spending more than 3 h on near work activities. Significant statistical differences were observed between the myopic and non-myopic groups in variables such as age, gender, BMI, parental myopia, time spent on near work activities, and area of residence (all, *p* < .0001) (Table 1).

**Table 1:** Characteristics of participants aged 13–18 years according to myopia and non-myopia.

	Total	Myopia	Non-myopia	*p*-value
Number, (%)	18,077	15,843 (87.64%)	2,234 (12.36%)	
Age (years)	15.05 ± 1.67^a^	15.07 ± 1.67	14.91 ± 1.66	<.0001^b^
Gender, *n* (%)				<.0001^c^
Male	9,346 (51.70%)	7,770 (49.04%)	1,576 (70.55%)	
Female	8,731 (48.30%)	8,073 (50.96%)	658 (29.45%)	
Refractive error (SE*) (D)	−3.41 ± 2.50	−3.90 ± 2.28	0.07 ± 0.45	<.0001
BMI** (kg/m^2^)	21.68 ± 4.04	21.62 ± 4.10	22.08 ± 3.53	<.0001
Parental myopia, *n* (%)				<.0001
Father	4,190 (23.18%)	3,674 (23.19%)	516 (23.10%)	
Mother	4,563 (25.24%)	3,703 (23.37%)	860 (38.50%)	
Both parents	3,828 (21.18%)	3,636 (22.76%)	222 (9.94%)	
Neither parent	5,496 (30.40%)	4,860 (30.68%)	636 (28.47%)	
Near work, *n* (%)				<.0001
≤1 h/day	1,024 (5.66%)	903 (5.70%)	121 (5.42%)	
1–2 h/day	4,591 (25.40%)	3,775 (23.83%)	816 (36.53%)	
3 h/day	4,231 (23.41%)	3,895 (24.58%)	336 (15.04%)	
≥4 h/day	8,231 (45.53%)	7,270 (45.89%)	961 (43.02%)	
Residence, *n* (%)				<.0001
Urban	12,664 (70.06%)	11,248 (71.00%)	1,416 (63.38%)	
Rural	5,413 (29.94%)	4,595 (29.00%)	818 (36.62%)	

SE*, spherical equivalent; BMI**, body mass index.

^a^ Mean ± standard deviation (SD).

^b^ Difference between two groups at *α* = 0.05 by ANCOVA test adjusted for age.

^c^ Difference between two groups at *α* = 0.05 by chi-square test.

### Distribution of refractive errors in adolescent participants

Among the adolescents aged 13–18 participating in this study, the prevalence of myopia was strikingly high at 87.6%, with high myopia accounting for 15.9% of these cases. An age-segmented analysis revealed that the prevalence of all types of myopia, including high myopia, did not exhibit substantial variation with increasing age. However, the mean of spherical equivalent (SE) for myopic group did progressively increase (Table 2).

**Table 2:** Prevalence and the spherical equivalent of refractive errors according to age.

Age (years)	Myopia (SE* ≤ −0.50 D)	High myopia (SE ≤ −6.00 D)	Emmetropia (−0.50 D < SE < +0.50 D)	Hyperopia (SE ≥ +0.50 D)
	*N* (%)			
13–14	5,586 (71.51%)	1,146 (14.67%)	946 (12.11%)	134 (1.72%)
15–16	4,296 (72.19%)	992 (15.90%)	468 (9.97%)	114 (1.94%)
17–18	3,087 (70.24%)	736 (16.75%)	548 (12.47%)	24 (0.55%)
Total	12,969 (71.74%)	2,874 (15.90%)	1,962 (10.85%)	272 (1.50%)
Age (years)	Myopia	High myopia	Emmetropia	Hyperopia
	Mean ± SD** (D)			
13–14	−2.93 ± 1.49	−7.40 ± 1.32	−0.02 ± 0.14	+0.16 ± 0.06
15–16	−3.19 ± 1.71	−7.58 ± 1.00	+0.04 ± 0.23	+1.20 ± 0.47
17–18	−3.31 ± 1.56	−7.51 ± 1.26	−0.29 ± 0.12	+0.88 ± 0.00
Total	−3.11 ± 1.59	−7.49 ± 1.21	−0.08 ± 0.21	+1.10 ± 0.32

SE*, spherical equivalent; SD**, standard deviation.

### Risk factors for myopia in adolescents

In a univariate logistic regression model adjusted for age and gender, several factors—including gender, age, BMI, parental history of myopia, daily hours spent on near work activities, and region of residence—were found to be associated with myopia in adolescents (Table 3). The risk of developing myopia was 2.51 times higher in female adolescents compared to male adolescents (95% CI, 2.28–2.76; *p* < .0001). Additionally, the risk of myopia decreased with increasing age (*p* < .0001). A positive correlation was also observed between an increase in BMI and the risk of myopia. Having both parents with myopia was associated with a 1.98-fold increase in the risk of developing myopia in their offspring (95% CI, 1.69–2.33; *p* < .0001). Furthermore, engaging in near work activities for 3 or more hours per day increased the risk of myopia by 1.61 times (95% CI, 1.29–2.01; *p* < .0001). Conversely, residing in rural areas was associated with a 0.68-fold reduction in the risk of myopia compared to living in urban settings (95% CI, 0.62–0.75; *p* < .0001).

**Table 3:** Association with risk factors in myopic adolescents aged 13–18 years using univariable logistic regression analysis.

	Myopia (*N* = 15,843)	*p*-value
	Adjusted OR* [95% CI**]^a^	
Gender		
Male	1	
Female	2.51 [2.28–2.76]	<.0001
Age (years)		
13–14	1	
15–16	0.85 [0.68–1.05]	<.0001
17–18	0.42 [0.28–0.62]	<.0001
BMI*** (kg/m^2^)		
<18.5	1	
18.5–24.9	0.40 [0.35–0.45]	<.0001
25–29.9	0.66 [0.56–0.77]	0.009
≥30	0.41 [0.32–0.52]	<.0001
Parental myopia		
Neither parent	1	
Father	0.89 [0.79–1.01]	0.110
Mother	0.47 [0.42–0.53]	<.0001
Both parents	1.98 [1.69–2.33]	<.0001
Near work		
≤1 h/day	1	
1–2 h/day	0.56 [0.46–0.69]	<.0001
3 h/day	1.61 [1.29–2.01]	<.0001
≥4 h/day	0.86 [0.70–1.06]	0.030
Residence		
Urban	1	
Rural	0.68 [0.62–0.75]	<.0001

OR*, odds ratio; CI**, confidence interval; BMI***, body mass index.

^a^ Adjusted for age and gender.

### Association between average daily intake of dietary nutrients and myopia

After adjusting for age and gender, we analyzed the covariance between the myopic and non-myopic groups for the intake of 14 major dietary nutrients. Statistically significant differences were found between the two groups for all 14 nutrients (Table 4). Both groups exhibited higher average daily intakes of nutrients like carbohydrates, proteins, fats, iron, sodium, vitamin B1, and niacin. However, the myopic group had significantly higher average daily intakes of protein, fat, cholesterol, calcium, iron, sodium, and vitamin B2 (all, *p* < .0001). Conversely, the non-myopic group consumed higher levels of nutrients like vitamin A, vitamin B1, niacin, and vitamin C, with the differences being statistically significant (niacin; *p* = 0.002, all others, *p* < .0001).

**Table 4:** Average daily intake of dietary nutrients in adolescents aged 13–18 years old according to myopia and non-myopia.

Nutrients	KDRI*	Myopia (*N* = 15,843)	Non-myopia (*N* = 2,234)	*p*-value^a^
Carbohydrate (g)	130	322.18 ± 0.45^b^	339.91 ± 1.21	<.0001
Protein (g)	25–60	84.74 ± 0.20	80.85 ± 0.53	<.0001
Fat (g)	25	64.99 ± 0.15	60.69 ± 0.41	<.0001
Cholesterol (mg)	300	349.68 ± 1.58	250.92 ± 4.24	<.0001
Calcium (mg)	600–1,000	500.51 ± 1.61	478.92 ± 4.33	<.0001
Phosphorus (mg)	550–1,200	1,158.32 ± 1.94	1,191.05 ± 5.20	<.0001
Iron (mg)	7–14	16.49 ± 0.07	15.57 ± 0.18	<.0001
Sodium (mg)	1,000–1,500	3,739.94 ± 9.93	3,536.66 ± 26.65	<.0001
Potassium (mg)	2,400–3,500	2,750.56 ± 7.19	3,238.07 ± 19.31	<.0001
Vitamin A (μgRE)	300–750	388.83 ± 5.43	704.59 ± 14.58	<.0001
Vitamin B1 (mg)	0.5–1.1	2.14 ± 0.01	2.45 ± 0.02	<.0001
Vitamin B2 (mg)	0.6–1.5	1.54 ± 0.00	1.38 ± 0.01	<.0001
Niacin (mg)	7–15	16.63 ± 0.05	17.04 ± 0.13	0.002
Vitamin C (mg)	45–90	82.38 ± 0.74	117.72 ± 1.99	<.0001

KDRI*, dietary reference intakes for Koreans.

^a^ Difference between two groups at *α* = 0.05 by ANCOVA test adjusted for age, gender, and energy.

^b^ Least Square Means ± standard error (SE).

To further explore the relationship between dietary nutrient intake and myopia, three models were employed for multivariate logistic regression analysis: Model 1 adjusted for age, gender, and BMI; Model 2 additionally adjusted for parental history of myopia; and Model 3 adjusted for age, gender, BMI, and time spent on near work activities. The analysis revealed that the dietary nutrient intake levels significantly influenced the prevalence of myopia among adolescents aged 13–18 years (Table 5). In Model 2, the prevalence of myopia was 1.88 times higher (95% CI, 1.71–2.07) for those with high carbohydrate intake, 1.54 times higher (1.40–1.69) for high protein intake, 1.80 times higher (1.64–1.98) for high cholesterol intake, 1.75 times higher (1.59–1.92) for high sodium intake, and 2.39 times higher (2.18–2.63) for high vitamin B2 intake (all, *p* < .0001). On the contrary, high vitamin C intake was associated with a 0.62-fold reduction (0.57–0.68) in myopia prevalence (*p* < .0001). Model 3 yielded similar results to Model 2. Specifically, high dietary intake of carbohydrates, protein, cholesterol, sodium, and vitamin B2 were significantly associated with increased myopia prevalence, whereas vitamin C intake was linked with a reduced prevalence. Consequently, the dietary nutrients that maintained a significant association with myopia, even after adjusting for potential confounding variables, included carbohydrates, proteins, cholesterol, calcium, iron, sodium, potassium, vitamin A, vitamin B1, vitamin B2, and vitamin C.

**Table 5:** Odds ratio of high intake of nutrients and having myopia in multivariable logistic regression analysis.

Nutrients	Model 1		Model 2		Model 3	
	OR* [95% CI**]^a^	*p*-value	OR [95% CI]^b^	*p*-value	OR [95% CI]^c^	*p*-value
Carbohydrate (g)	1.90 [1.72–2.09]	<.0001	1.88 [1.71–2.07]	<.0001	1.88 [1.70–2.07]	<.0001
Protein (g)	1.48 [1.35–1.62]	<.0001	1.54 [1.40–1.69]	<.0001	1.51 [1.38–1.66]	<.0001
Fat (g)	0.89 [0.81–0.98]	0.017	0.91 [0.83–1.00]	0.054	0.92 [0.83–1.00]	0.061
Cholesterol (mg)	1.74 [1.59–1.91]	<.0001	1.80 [1.64–1.98]	<.0001	1.78 [1.62–1.95]	<.0001
Calcium (mg)	1.27 [1.16–1.39]	<.0001	1.32 [1.20–1.44]	<.0001	1.33 [1.21–1.46]	<.0001
Phosphorus (mg)	0.95 [0.87–1.04]	0.256	0.99 [0.90–1.08]	0.779	0.98 [0.90–1.08]	0.702
Iron (mg)	1.35 [1.23–1.48]	<.0001	1.39 [1.27–1.53]	<.0001	1.37 [1.25–1.50]	<.0001
Sodium (mg)	1.72 [1.57–1.89]	<.0001	1.75 [1.59–1.92]	<.0001	1.79 [1.63–1.97]	<.0001
Potassium (mg)	1.33 [1.21–1.46]	<.0001	1.38 [1.26–1.51]	<.0001	1.35 [1.23–1.48]	<.0001
Vitamin A (μgRE)	1.10 [1.01–1.20]	0.039	1.16 [1.06–1.28]	0.001	1.12 [1.02–1.22]	0.018
Vitamin B1 (mg)	1.17 [1.06–1.28]	0.001	1.16 [1.06–1.27]	0.002	1.21 [1.10–1.33]	<.0001
Vitamin B2 (mg)	2.28 [2.08–2.51]	<.0001	2.39 [2.18–2.63]	<.0001	2.36 [2.15–2.60]	<.0001
Niacin (mg)	0.92 [0.84–1.01]	0.070	0.93 [0.85–1.02]	0.144	0.93 [0.85–1.02]	0.108
Vitamin C (mg)	0.62 [0.56–0.68]	<.0001	0.62 [0.57–0.68]	<.0001	0.64 [0.58–0.70]	<.0001

OR*, odds ratio; CI**, confidence interval.

^a^ Adjusted for age, gender, and BMI.

^b^ Adjusted for age, gender, BMI, and parental myopia.

^c^ Adjusted for age, gender, BMI, and near-work time.

## Discussion

This study found that the prevalence of myopia among Korean adolescents between the ages of 13 and 18 is 87.6%. This rate is notably higher than the 73.0% reported for the age group of 12–18 by the National Health and Nutrition Examination Survey conducted from 2008 to 2012 (6). These findings indicate a continuous increase in myopia prevalence. For international context, the prevalence rates are 70.9% in China for those aged 6–18 (19), 42.7% in France for those aged 10–19 (20), and 7% in South Africa for the age group of 13–18 (21). This underscores that the rate of myopia in Korean adolescents is exceptionally high.

The study identified several factors that influence the prevalence of myopia in adolescents, including age, gender, BMI, family history, near work hours, and residential area. These results corroborate findings from numerous prior studies (19, 22–26). Notably, the risk of developing myopia was found to be significantly higher among female adolescents and individuals with a family history of the condition. Although the overall prevalence did not vary significantly by age, we observed that the refractive power associated with myopia increased with age. This supports existing research that suggests myopia continues to progress through adolescence (27).

The objective of this study was to investigate the association between dietary nutrients and myopia prevalence among Korean adolescents aged 13–18. While previous research has focused on specific nutrients like omega-3 fatty acids, vitamin A, and sodium (14, 28, 29), our study broadened the scope to include 14 different macro- and micronutrients. We found that dietary intake of carbohydrates, protein, cholesterol, sodium, vitamin A, vitamin B2, and vitamin C was associated with the prevalence of myopia.

The average carbohydrate intake among adolescents in this study exceeded the recommended daily intake and was found to be linked to higher myopia prevalence. This aligns with Cordain et al.'s hypothesis that refined carbohydrate consumption may contribute to the development of myopia through hyperinsulinemia, affecting the eye's axial length (30). Recent research has provided supporting evidence for this carbohydrate-myopia connection (31, 32). For instance, Berticat et al. found a correlation between higher intake of refined carbohydrates and increased prevalence of myopia in a study of French children and adolescents (31). Liu et al. also found that a diet rich in whole grains acted as a protective factor against myopia in Chinese children (33).

Recent studies present conflicting evidence concerning the role of dietary nutrients in myopia. For example, our research found a direct correlation between higher cholesterol intake and increased risk of myopia. However, a study involving 851 Chinese adolescents in Singapore indicated that neither saturated fats nor cholesterol were conclusively related to myopia, despite their association with longer axial length (32). Similarly, while our study showed a significant relationship between vitamin A and myopia risk, a study by Ng et al. involving Australian adolescents found no such link (28). In addition, this study found that among the assessed nutrients, vitamin B2 posed the highest risk for myopia while vitamin C appeared protective. These results are at odds with a study involving Irish children and adolescents, which identified vitamin D as the only nutrient significantly associated with myopia (34). In Korea, sodium consumption is notably high—about half the population consumes more than twice the recommended daily intake (35, 36). In our study, both myopic and non-myopic groups exceeded this intake, and higher sodium consumption correlated with increased myopia risk.

Research by Yin et al. on Chinese children highlighted dietary patterns characterized by higher consumption of meat, aquatic products, eggs, legumes, milk, dairy, vegetables, fruits, grains, and potatoes as lowering the risk of myopia (37). Interestingly, consumption of snacks and beverages showed no association with myopia. While our study did not focus on sugar-related nutrients, Ren et al. found that the prevalence of myopia among Chinese children was linked to consumption of processed foods containing sugar, such as cakes and canned fruits (38). Data from the Korean Adolescent Health Behavior Survey indicates high consumption rates of carbonated and sweetened drinks among adolescents (39, 40), suggesting that further studies on saccharide-related nutrients and their relationship with myopia may be warranted.

The dietary habits of individuals vary significantly across cultures, influenced by regional, environmental, and traditional factors. This diversity in eating patterns is particularly evident when comparing Western and Eastern countries. Western diets often emphasize higher intakes of processed foods, red meats, and dairy products, while Eastern diets, especially those in Asian countries, are characterized by higher consumption of rice, vegetables, fish, and soy products (41, 42). These fundamental differences in diet composition can significantly impact health outcomes, including conditions like myopia. The nutritional content, such as the balance of vitamins, minerals, and other nutrients, varies widely between these diets (43). For instance, the prevalence of myopia in different regions might be influenced by the varying levels of specific nutrients like omega-3 fatty acids, vitamin A, and refined carbohydrates, which are distributed differently in Western and Eastern diets. Understanding these cultural differences in dietary habits is crucial for comprehensively examining the role of nutrition in the development and progression of myopia and other health conditions. To address this, future research could benefit from a comparative analysis involving similar studies conducted in various regions of the world. This would allow for a more nuanced understanding of how regional dietary habits, in conjunction with genetic factors, contribute to the prevalence of myopia. Such comparative studies could uncover patterns that are consistent across different cultural contexts, thereby strengthening the generalizability of the findings.

In addition, in our study, we observed a higher prevalence of myopia in urban areas, which is consistent with existing literature (44–46). Urban environments are often associated with a higher consumption of convenience foods, which are typically processed and high in carbohydrates and sodium. In contrast, rural diets might include more fresh produce and traditional foods, potentially leading to different nutritional profiles. This urban-rural divide in dietary habits could be a significant factor in understanding the environmental contributions to myopia. Future studies could benefit from stratifying data based on the urban-rural residence of participants. Such stratification would provide clearer insights into how different living environments, with their respective dietary habits, impact the prevalence and development of myopia.

While our study makes a valuable contribution by exploring the possible link between dietary nutrient intake and myopia in a large sample of Korean adolescents, it comes with several limitations. Firstly, the cross-sectional nature of our study design hampers the ability to establish a causal relationship between diet and myopia. Longitudinal studies are needed for more robust conclusions. Secondly, the use of self-reported data from the National Health and Nutrition Examination Survey introduces the potential for recall bias or inaccuracies. Future studies employing more precise dietary assessment methods, such as food diaries or frequency questionnaires, are advisable. Thirdly, the disproportionate number of subjects in the myopic group compared to the non-myopic group could potentially skew the average dietary nutrient intake, particularly for the non-myopic group. Lastly, we did not account for other possible confounding factors like outdoor activity, screen time, and genetic predisposition, which could influence myopia. Our study is observational, hence it cannot definitively establish that dietary nutrient intake causes myopia. Although no diet is currently recommended specifically for myopia prevention, based on our findings, it might be prudent for adolescents to maintain a balanced diet. This could include a variety of whole grains, fruits, and vegetables while minimizing consumption of processed foods high in carbohydrates, sodium, and cholesterol.

Our study identified several dietary nutrients— including carbohydrates, protein, cholesterol, calcium, iron, sodium, potassium, vitamin A, vitamin B1, and vitamin B2—as significantly related to the prevalence of myopia among Korean adolescents. Interestingly, vitamin C intake appeared to lower the risk. While our study suggests that nutrient intake may play a role in the high prevalence of myopia, these findings should be interpreted cautiously due to the study's limitations. Further investigations are needed to better understand the role and impact of dietary nutrients on the high prevalence of myopia in this demographic.

## Data Availability

The original contributions presented in the study are included in the article/Supplementary Material, further inquiries can be directed to the corresponding author.
